# High accuracy of an ELISA test based in a flagella antigen of *Leishmania* in serodiagnosis of canine visceral leishmaniasis with potential to improve the control measures in Brazil – A Phase II study

**DOI:** 10.1371/journal.pntd.0006871

**Published:** 2018-10-26

**Authors:** Lairton Souza Borja, Lívia Brito Coelho, Matheus Silva de Jesus, Artur Trancoso Lopo de Queiroz, Paola Alejandra Fiorani Celedon, Nilson Ivo Tonin Zachin, Edimilson Domingos Silva, Antônio Gomes Pinto Ferreira, Marco Aurélio Krieger, Patrícia Sampaio Tavares Veras, Deborah Bittencourt Mothé Fraga

**Affiliations:** 1 Laboratório de Interação Parasito-Hospedeiro e Epidemiologia, Instituto Gonçalo Moniz–FIOCRUZ, Salvador, Bahia, Brazil; 2 Centro de Integração de Dados e Conhecimentos para Saúde, Instituto Gonçalo Moniz–FIOCRUZ, Salvador, Bahia, Brazil; 3 Instituto de Biologia Molecular do Paraná -IBMP, Curitiba, Paraná, Brazil; 4 Instituto Carlos Chagas–FIOCRUZ, Curitiba, Paraná, Brazil; 5 Instituto de Tecnologia em Imunobiológicos, Bio-Manguinhos, Rio de Janeiro, RJ, Brasil; 6 Instituto Nacional de Ciência e Tecnologia em Doenças Tropicais, INCT—DT, Bahia, Brazil; 7 Escola de Medicina Veterinária, Universidade Federal da Bahia, Salvador, Bahia, Brazil; Saudi Ministry of Health, SAUDI ARABIA

## Abstract

**Background:**

Canine Visceral leishmaniasis (CVL) is a serious public health problem, thus for its control, the Ministry of Health in Brazil recommends the rapid diagnosis and euthanasia of seropositive dogs in endemic areas. Therefore, our group had previously selected six recombinant proteins (rLci1, rLci2, rLci4, rLci5, rLci8, and rLci12) due to their high potential for CVL diagnostic testing. The present study aims to produce an immunodiagnostic test using the aforementioned antigens, to improve the performance of the diagnosis of CVL recommended by Brazilian Ministry of Health.

**Methodology/Principal findings:**

To evaluate the recombinant proteins in the serological assays, positive and negative samples were selected based on parasitological test (culture) and molecular test (qPCR) of splenic aspirate. Initially, we selected 135 dog serum samples, 73 positives (symptomatic and asymptomatic) and 62 negatives to screen recombinant proteins on ELISA platform. Then, for rLci5 ELISA validation, 361 serum samples collected in a cross-sectional study were selected, being 183 positives (symptomatic and asymptomatic) and 178 negatives. In the screening of the recombinant proteins, rLci5 was the only protein to present a performance statistically higher than the performance presented by EIE^-^LVC test, presenting 96% (IC 95%; 85–99%) vs. 83% (IC 95%; 69–92%) of sensitivity for symptomatic dogs, 71% (IC 95%; 49–97%) vs. 54% (IC 95%; 33–74%) for asymptomatic dogs and 94% (IC 95%; 83–99%) vs, 88% (IC 95%; 76–95% of specificity. Thus, the rLci5 protein was selected to compose a final ELISA test. Validation of rLci5 ELISA showed 87% (IC 81–91%) of sensitivity, 94% (IC 95%; 90–97%) of specificity and 90% accuracy. Testing the EIE^-^LVC with the same validation panel, we observed a lower performance when compared to ELISA rLci5 (sensitivity of 67% (IC 95%; 59–74%), specificity of 87% (IC 95%; 81–92%), and accuracy of 77%). Finally, the performance of current CVL diagnostic protocol recommended by Brazilian Ministry of Health, using DPP^-^LVC as screening test and EIE^-^LVC as confirmatory test, was compared with a modified protocol, replacing EIE^-^LVC by rLci5 ELISA. The current protocol presented a sensitivity of 59% (IC 95%; 52–66%), specificity of 98% (IC 95%; 95–99%) and accuracy of 80% (IC 95%; 76–84%), while the modified protocol presented a sensitivity of 71% (IC 95%; 63–77%), specificity of 99% (IC 95%; 97–100%) and accuracy of 86% (IC 95%; 83–89%).

**Conclusion:**

Thus, we concluded that rLci5 ELISA is a promising test to replace EIE^-^LVC test and increase the diagnostic performance of CVL in Brazil.

## Author summary

Visceral leishmaniasis is a tropical disease caused by the protozoan parasite *Leishmania infantum*, which is transmitted to humans through the bite of infected sand flies. Infected dogs are considered the main urban reservoir of parasite. Identification and euthanasia of infected dogs is one of the main strategies for VL control recommended by the Brazilian Ministry of Health. Hence, to improve the efficacy of control measures, it is essential an accurate diagnose of naturally infected dogs in endemic areas. Herein, we sought to produce a new immunodiagnostic test using recombinant antigens of *Leishmania infantum* to improve the performance of the diagnosis of CVL recommended by Brazilian Ministry of Health.

## Introduction

Visceral Leishmaniasis (VL) caused by *Leishmania infantum* (syn *Leishmania chagasi*) in Brazil, is a zoonotic disease and infected dogs are considered the main urban reservoir [1–4]. Diagnosis and euthanasia of infected dogs are one of the main strategies for VL control recommended by the Brazilian Ministry of Health. Thus, accurate diagnosis is essential to correctly identify animals infected with *L*. *infantum* [5,6]. In 2012, a rapid immunochromatographic test (DPP-LVC) based on the rK28 protein has recently become the preferred diagnostic method for screening in Brazil, followed by ELISA (EIE-LVC) as a confirmatory test. Recent studies have shown that EIE-LVC presents a highly variable sensitivity and specificity in diagnosing canine visceral leishmaniasis (CVL), varying between 72% to 97% for sensitivity, and 26% to 84% for specificity [7,8]. Additionally, EIE-LVC are known to present a low sensitivity in the detection of asymptomatic dogs [9,10], indicating current diagnostic tests of CVL need to be replaced. For improving VL control measures, the identification of novel recombinant antigens may contribute to enhance test sensitivity and specificity.

Therefore, six recombinant antigens (Lci1A, Lci2B, Lci4, Lci5, Lci8 and Lci12) were previously selected from a cDNA and a genomic library of *Leishmania infantum* by our group, as described by Oliveira et al. [11], Teixeira et al. [12] and Magalhães et al. [13], and screened by a Multi-antigen print immunoassay (MAPIA) technique [14] due to their strong potential as candidates in diagnostic testing. Previous results obtained by Oliveira et al. [11] and Souza et al. [15] demonstrated by ELISA the potential of some of these recombinant antigens for the development of a diagnostic test. Moreover, two of these proteins was already tested in a DPP platform, an immunochromatographic rapid test prototype based on the dual path platform. The sensitivity of the DPP Prototype with Lci1A and Lci2B evaluated in a multi-centric study was 87%, similarly to the 88% of sensitivity from the test currently provided by the Brazilian Minister of Health, DPP-LVC [16]. The present study aimed to evaluate the accuracy of the recombinant antigens (Lci1A, Lci2B, Lci4, Lci5, Lci8 and Lci12) of *Leishmania infantum* for the serodiagnosis of dogs infected by *L*. *infantum*, which were selected by its highest accuracy to compose a final ELISA test, and validate the use of this test to improve the diagnostic protocol for CVL currently in use by Brazilian Minister of Health in Brazil.

## Methods

### Ethical considerations

All serological samples were obtained according to the procedures approved by the Institutional Review Board for Animal Research (CEUA, protocol no. 03/2013) at the Federal University of Bahia in Salvador, Bahia-Brazil.

### *Leishmania infantum* antigens selection

A set of six recombinant *L*. *infantum* antigens (rLci1A, rLci2B, rLci4, rLci5, rLci8, rLci12) was previously selected from a cDNA and a genomic library of *Leishmania infantum* based on antibody reactivity using a pool of sera from culture-positive dogs and human patients with VL [11,16]. These recombinant antigens were screened using a multi-antigen print immunoassay (MAPIA) [14] technique to verify potential as viable candidates in diagnostic testing.

### Antigen production and purification

Antigen production and purification was carried out at the Instituto Carlos Chagas (ICC-Paraná), Universidade Federal de Minas Gerais (UFMG) and Instituto Oswaldo Cruz (IOC-Rio de Janeiro). *Escherichia coli* BL21 (DE3) *pLysS* (Invitrogen) was transformed with pRSET plasmids (Invitrogen) containing the Lci1, Lci2, Lci4, Lci5, Lci8 or Lci12 *L*. *infantum* gene insert [11]. Transformed bacteria were grown in Lysogeny broth medium and induced using 0.1 mM of isopropyl β- d -thiogalactoside overnight at 15°C to express Lci1, Lci4, Lci5, or at 37°C for 3h for Lci2 and Lci12 expression. Affinity chromatography was used to purify the proteins rLci1, rLci2, rLci5 and rLci8 from the soluble extract of proteins, while the crude extract was used to purify the rLci4 and rLci12 proteins, both using a HisTrap HP column (GE Healthcare, Piscataway, NJ) connected to an AKTAprime chromatography system (GE Healthcare, USA). rLci2 and rLci8 were submitted to a second purification step by ion-exchange chromatography using a Hitrap Q HP column (GE Healthcare, Piscataway, NJ) connected to an AKTAprime chromatography system.

### Serum samples

#### Screening of recombinant antigens

To identify which recombinant antigens offered the highest performance in CVL diagnosis amongst rLci1A, rLci2B, rLci4, rLci5, rLci8 and rLci12, 135 reference samples were selected from a characterized serum bank at the Laboratório de Interação Parasito-Hospedeiro e Epidemiologia (FIOCRUZ-BA). Reference samples obtained from males and females of naturally infected and non-infected dogs with variable ages ranging from 8 months to 6 years from endemic areas in the state of Bahia (Camaçari, Dias d’Avila and Lauro de Freitas) and non-infected dogs with variable ages from the non-endemic area of Pelotas (Rio Grande do Sul) were selected from a characterized serum bank at the Laboratório de Interação Parasito-Hospedeiro e Epidemiologia. All the samples were selected according to the results of culture and qPCR [8,17] from a splenic aspirate of dogs. Positive ones presented positive results in culture or qPCR, and negative ones presented negative results in both diagnostic tests. The qPCR was performed as previously described by Solcà et al. [17]. The taqMan-MGB probe and PCR primers were designed to target conserved DNA regions of the kinetoplast minicircle DNA from L. infantum to obtain a 120-bp amplicon. The qPCR amplification protocol employed the following primers: forward primer 5'-AACTTTTCTGGTCCTCCGGGTAG-3' (Leish-1) and reverse primer 5'-ACCCCCAGTTTCCCGCC-3' (Leish-2), both at a final concentration of 900 nM. A fluorogenic probe 5’-AAAAATGGGTGCAGAAAT-3’ was used for detection, synthesized using a FAM reporter molecule attached to the 5’ end, as well as a MGB-NFQ quencher linked to the 3’-end (Perkin-Elmer Applied Biosystems) at a final concentration of 200 nM. The reference sample panel consisted of 73 serum samples from naturally infected dogs and 51 samples from negative dogs. *L*. *infantum*-positive samples were further divided into sets of 49 samples originating from symptomatic dogs and 24 from asymptomatic dogs.

*L*. *infantum*-infected dogs had been previously classified as asymptomatic or symptomatic based on a score calculated according to the intensity of the following evaluated clinical signs: emaciation, alopecia, anemia, conjunctivitis, dehydration, dermatitis, erosion, ulcerations, lymphadenopathy and onychogryphosis. Animals were classified as asymptomatic when presenting a score of 0–3, or symptomatic when classified with a score higher than three.

#### Validation of the ELISA rLci5 test

The sample size needed to validate the results of the rLci5 ELISA was calculated with a 95% confidence interval, an expected sensitivity and specificity of 90%, and an absolute error of 5%. Based on these parameters, we used the Open Epi program [18] to calculate a sample size for the validation sera panel, which consisted of 183 sera from naturally infected dogs from endemic areas of Bahia (123 symptomatic dogs and 60 asymptomatic dogs), 178 samples of negative dogs also from endemic areas in Bahia, and 31 samples of dogs experimentally infected with *Trypanosoma cruzi* in acute phase of the disease. The animals experimentally infected with *T*. *cruzi* were born at a kennel, treated with anthelminthic, immunized against more common canine infectious pathogens and protected against leishmaniasis infection. The samples were obtained from a serum bank at Laboratório de Interação Parasito-Hospedeiro e Epidemiologia (FIOCRUZ-BA), and the selection process was based solely on previously obtained results from culture and quantitative PCR using splenic aspirate samples (i.e. the previously confirmed serological status was not considered, in order to avoid biased performance results).

### ELISAs

#### Screening of recombinant antigens

To identify the recombinant antigens that offered the highest performance, a recombinant antigen in-house ELISA was performed as previously described by Oliveira et al. [11] with slight modifications for standardization purposes. Protein samples were diluted in coating buffer (15 mM Na_2_HCO_3_, 28 mM NaHCO_3_; pH 9.6) at the following concentrations: 0.3 μg/ml of Lci1, 0.15 μg/ml of Lci2, 0.5 μg/ml of Lci4, 0.5 μg/ml of Lci5, 0.5 μg/mL of rLci8 and 0.1 μg/ml of Lci12.The dilutions of each recombinant antigen were plated on 96-well microtiter plates (only one recombinant antigen per well) and incubated overnight at 4°C. A solution with 0.15 M PBS (pH 7.2) containing 0.05% Tween 20 and 4mg/mL of bovine albumin serum (BSA) was used to block free sites in the wells. Plates were subsequently incubated with reference serum samples at a ratio of 1:800 (canine serum) at 37°C for 1 h. Wells were then washed with 0.15 M PBS (pH 7.4) containing 0.05% Tween 20 and peroxidase AffiniPure Rabbit Anti-Dog IgG (H+L) (diluted at 1:8000) (Jackson Imunnoresearch, West Grove, PA) was added, followed by incubation for 1 h at 37°C. Enzymatic activity was detected by 0.01% hydrogen peroxide and 0.01% 3,3′,5,5′-Tetramethylbenzidine (Sigma- Aldrich) in 0.1 M phosphate-citrate buffer (pH 5.0). The reaction was stopped with 50 μL of 4 M H_2_SO_4_, and plates were then read in a spectrophotometer using a 450 nm filter. Optical density (OD) cutoff values pertaining to optimal sensitivity and specificity were defined using a Receiver Operator Curve (ROC curve) considering the OD samples of negative and positive samples in each plate. It was also performed according methodology above an ELISA using a combination of 0.5 μg/ml of rLci5 and 0.01 μg/ml rLci12.

#### Validation of rLci5 ELISA

After identifying the recombinant antigen with the best performance, an ELISA kit employing only that specific protein, Lci5, was tested (rLci5 ELISA) and results were compared to the performance of the DPP-LVC and EIE-LVC protocols. The recombinant antigen was diluted in the coating buffer at a concentration of 0.25 μL/mL, 100 μL were added to the plate and then incubated overnight at 4°C. Plates were blocked and stored after adding WellChampion solution (Kem-En-Tec Diagnostics), and enzymatic activity was detected using TMB PLUS2 (Kem-En-Tec Diagnostics).

For comparison purposes, all diagnostic test procedures involving the EIE-LVC and DPP-LVC diagnostic tests were performed. These tests were carried out in accordance with manufacturer recommendations (Bio-Manguinhos/FIOCRUZ).

### Performance evaluation of the CVL diagnosis protocol

The performance of the CVL diagnosis protocol (DPP-LVC and EIE-LVC) recommended by Brazilian Ministry of Health was evaluated after rLci5 ELISA evaluation. Validation sera panel was tested using DPP-LVC and EIE-LVC tests. As recommended by the diagnostic protocol, DPP-LVC was used as screening test and EIE-LVC as confirmatory test (current protocol). Using the same sera sample panel, the performance of an alternative protocol employing rLci5 ELISA as a confirmatory test in place of EIE-LVC was evaluated and compared to the performance of the protocol currently recommended by the Ministry of Health.

### Statistical analysis

All diagnostic testing was performed under blinded conditions, which means that test readers interpreted the results obtained from each diagnostic technique for a given sample without knowledge of the results of other tests. Data were encoded, analyzed and presented using scatter plot graphing software (GraphPad Prism version 6, San Diego-CA, USA). All analyses were two-tailed, and a p-value of less than 5% was considered significant (p < 0.05). For the recombinant antigens ELISA, the cut-off pertaining to optimal sensitivity and specificity was established using a Receiver Operator Curve (ROC curve), while the EIE-LVC cut-off was obtained in accordance with the manufacturer’s recommendation (i.e. twice the average of the negative control). All results were expressed by plotting the obtained values in an index format representative of the ratio between a given sample’s OD and the cut-off OD pertaining to each microplate, referred to as reactivity index (RI), with all results <1.00 considered negative. Each serodiagnostic test was evaluated with respect to sensitivity, specificity, area under the curve (AUC) and accuracy. For comparisons among the diagnostic tests, differences were detected using the McNemar test and considered statistically significant when p < 0.05. Diagnostic odds ratio was calculated to show the ratio of the odds of disease in test positives relative to the odds of disease in test negatives. Confidence intervals (CI) were calculated using a confidence level of 95%.

## Results

### Recombinant antigen screening

**Fig 1:**
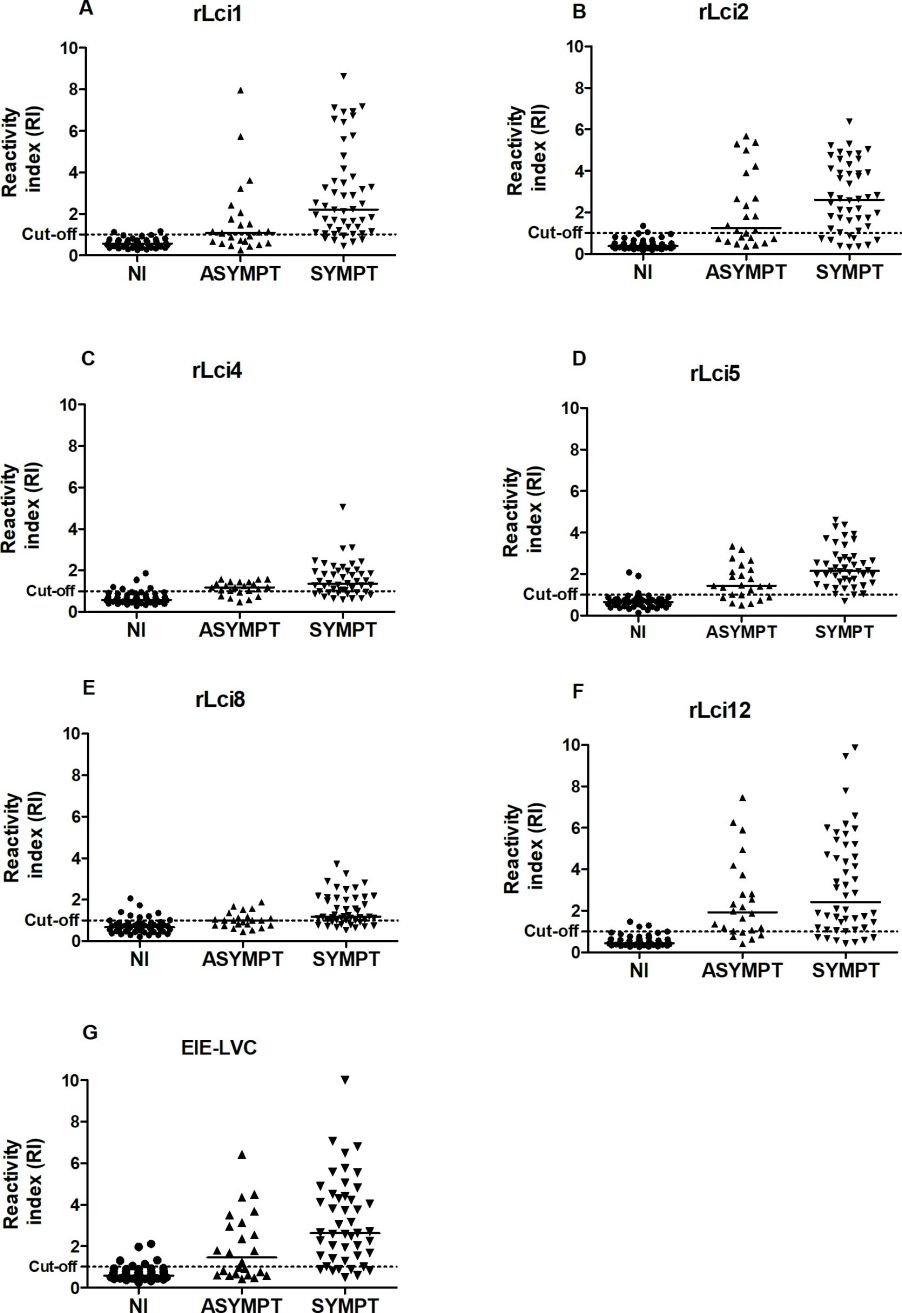
Diagnostic performance of recombinant antigens in an ELISA protocol and EIE-LVC. Reactivity index results obtained under *Leishmania* recombinant antigen ELISA, rLci1 (A), rLci2 (B), rLci4 (C), rLci5 (D), rLci8 (E), rLci12 (F) and the EIE-LVC (G) using serum from non-infected animals (NI), infected asymptomatic dogs (ASYMPT) and infected dogs presenting symptoms (SYMPT).

Each one of the following recombinant antigens of *Leishmania*, rLci1, rLci2, rLci4, rLci5, rLci8 and rLci12, was evaluated in an ELISA protocol to identify which of them offered highest performances in comparison to EIE-LVC, the confirmatory CVL diagnostic test recommended by Brazilian Ministry of Health. Fig 1 illustrates the performance parameters and reactivity index (RI) distributions obtained by ELISA using the recombinant antigens in addition to EIE-LVC. rLci1, rLci2 and rLci12 presented the highest RI differences between positive and negative samples. When testing samples from symptomatic dogs in comparison to asymptomatic animals, higher RI results were obtained using rLci1, rLci4, rLci5, rLci8 and the EIE-LVC test. None of these antigens demonstrated potential for use as a serological marker capable of differentiating a given animal’s clinical status based on RI differences.

The diagnostic performance of each recombinant antigen under ELISA, in addition to the EIE-LVC test, are summarized in Table 1. The recombinant antigens offering the best performance with regard to CVL diagnosis were rLci5 (92% accuracy), rLci12 (87% accuracy), rLci1 and rLci2 (84% accuracy), rLci4 (81% accuracy) and rLci8 (75% accuracy). Moreover, rLci5 ELISA also offered the greatest sensitivity with respect to detecting symptomatic dogs, in addition to the second highest sensitivity regarding asymptomatic dogs. Comparisons between the sensitivity results showed that rLci5 ELISA offered a statistically significant higher sensitivity in comparison to EIE-LVC (p < 0.05; OR = 2.6; CI: 1.02–7.28) (Table 1).

**Table 1:** Diagnostic performance of six ELISA assays employing recombinant *Leishmania* antigens to diagnose CVL using a reference sample panel, compared to EIE-LVC.

ELISA	Sensitivity (CI 95%; N = 73)	Sensitivity		Specificity (CI 95%; N = 51)	Accuracy (CI 95%)
		Asymptomatic dogs (CI 95%; N = 24)	Symptomatic dogs (CI 95%; N = 49)		
rLci1	77 (66–86%)	54 (33–74%)	88 (76–95%)	96% (86–99%)	84%
rLci2	74 (62–83%)^a^^b^	63 (41–81%)	81 (67–91%)	96% (86–100%)	84%
rLci4	73 (61–83%)	70 (47–87%)	72 (57–84%)	91% (80–97%)	81%
rLci5	89 (79–95%)^a^^b^	71 (49–97%)	96% (85–99%)	94% (83–99%)	92%
rLci8	69 (56–79%)	48 (27–69%)	74% (0–86%)	85% (73–93%	75%
rLci12	82 (71–90%)	79 (58–93%)	83% (70–93%)	92% (81–98%)	87%
EIE-LVC	73 (61–83%) ^b^	54 (33–74%)	83% (69–92%)	88% (76–95%)	79%

^a^ p<0.001; OR = 4.25 (CI 95% 1.4–17.4)–Comparison of sensitivity among recombinant antigens and EIE-LVC as evaluated by McNemar’s test

^b^ p<0.05; OR = 2.57 (CI 95% 1.02–7.28)–Comparison of sensitivity among recombinant antigens and EIE-LVC as evaluated by McNemar’s test

**Fig 2:**
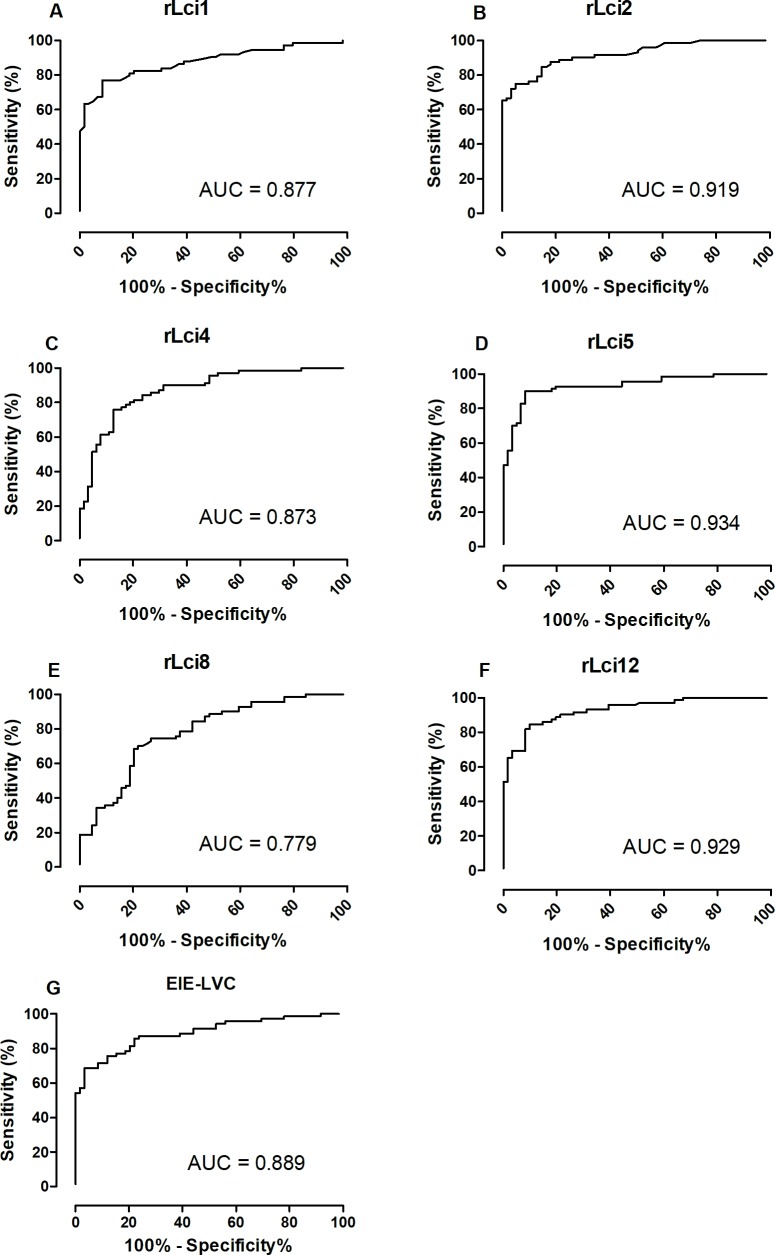
ROC curve analysis of the area under the curve (AUC), considering the results from ELISA employing *Leishmania* recombinant antigens, as well as EIE-LVC. The ROC curve obtained from antigens rLci1 (A), rLci2 (B), rLci4 (C), rLci5 (D), rLci8 (E), rLci12 (F) and EIE-LVC test (G) was established using serum samples of negative and positive samples in each plate.

ROC curve analysis of the area under the curve (AUC) (Fig 2) confirmed the superior performance of the rLci5 ELISA protocol (AUC = 0.934) in comparison to the rLci12 ELISA (AUC = 0.929) and EIE-LVC (AUC = 0.889) tests.

Recombinant antigen screening results identified rLci5 as the *Leishmania* protein offering the best CVL diagnostic performance for use in an ELISA commercial kit. Validation results for the rLci5 ELISA kit confirmed higher sensitivity (87%) than both EIE-LVC (67%), the confirmatory test recommended by Ministry of Health in Brazil, and DPP-LVC (74%), the screening test currently endorsed by the Brazilian Ministry of Health (Table 2). Additionally, rLci5 ELISA presented higher sensitivity in the detection of symptomatic dogs (85%) and asymptomatic dogs (93%) than either DPP-LVC (70% and 87%) or EIE-LVC (66% and 69%).

**Table 2:** Comparison of CVL diagnostic test performance using a validation serum panel.

Tests	Sensitivity (CI 95%; N = 183)	Sensitivity		Specificity (CI 95%; N = 178)	Cross-reactivity	LR+	LR-	Accuracy (CI 95%; N = 392)
		asymptomatic dogs (N = 60 (CI 95%)	symptomatic dogs (N = 123 (CI 95%)					
**ELISA rLci5 kit**	87 (81–91%)^a^^b^	83 (71–92%)	89 (82–94%)	94 (90–97%)	11/31 (35%)	14.5	0.14	90 (87–93%)
**DPP-LVC**	74 (67–80%)^a^	75 (62–85%)	73 (64–80%)	94 (89–97%)	03/31 (10%)	12.3	0.28	84 (80–88%)
**EIE-LVC**	67 (59–74%)^b^	57 (43–69%)	72 (63–80%)	87 (81–92%)	12/27* (44%)	5.1	0.38	77 (73–81%)

^a^ p<0.005; OR = 2.6 (CI 95% 1.40–5.01) McNemar Test

^b^ p<0,05; OR = 2.04 (CI 95% 1.21–3.52) McNemar Test

*Results from four samples were considered indeterminate and were not included in this analysis

LR+ (Positive likelihood ratio) and LR- (Negative likelihood ratio)

Stratification of the validation panel considering only samples with positive culture results with positive or negative qPCR (155 dogs) indicated slightly higher test sensitivity for rLci5 ELISA (95%), followed by 83% for DPP-LVC and 78% for EIE-LVC. However, when evaluating sera from dogs with only positive qPCR results (24 dogs), test sensitivity decreased drastically: 42% for Lci5 ELISA, 19% for DPP-LVC and 11% for EIE-LVC (Table 3). With regard to test cross-reactivity using serum samples from dogs experimentally infected with *Trypanosoma cruzi*, DPP-LVC presented the highest specificity (90%), followed by rLci5 ELISA (65%) and EIE-LVC (56%).

**Table 3:** Sensitivity of ELISA rLci5, DPP-LVC and EIE-LVC according to different previous diagnostic test results of the samples from validation serum panel.

Previous diagnostic test results (n = 183)	Sensitivity		
	rLci5 ELISA	DPP-LVC	EIE-LVC
**Culture positive and qPCR positive or negative** *	147/155 (95%)	128/154^1^ (83%)	121/155 (78%)
**qPCR positive and culture positive or negative**	155/179 (87%)	131/176 (74%)	120/170 (71%)
**qPCR positive only**	12/28 (42%)	05/27^2^ (19%)	03/27(11%)
**Culture positive only**	04/04 (100%)	04/04 (100%)	04/04 (100%)

*4 samples selected were positive only in culture

^1^01 samples were not evaluated in **DPP**^**-**^**LVC**

^2^01 sample were not evaluated in **DPP**^**-**^**LVC**

**Fig 3:**
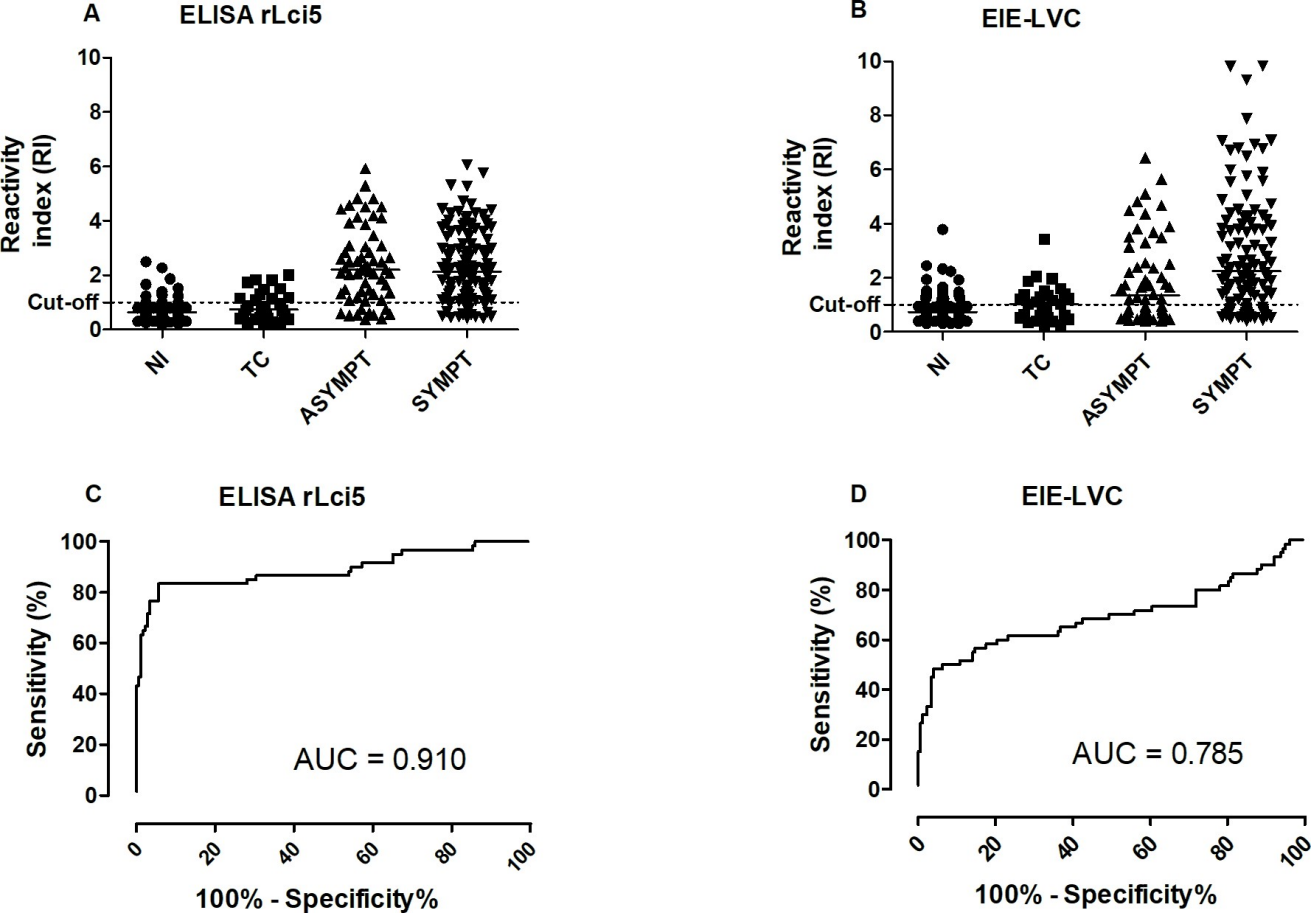
Evaluation of the reactivity index and AUC obtained for the rLci5 ELISA and EIE-LVC tests using the validation serum panel to diagnose CVL. Reactivity index and AUC results obtained under rLci5 ELISA (A, C) and EIE-LVC (B, D) using serum from non-infected animals (NI), animals experimentally infected with *T*. *cruzi* (TC), infected asymptomatic dogs (ASSYMPT) and infected dogs presenting symptoms (SYMPT).

Using the validation serum panel, a comparison of diagnostic performance assessing reactivity index and the area under the curve (AUC) revealed superior performance by Lci5 ELISA (AUC = 0.910) compared to EIE-LVC (AUC = 0.785) (Fig 3), despite the higher RI values obtained under EIE-LVC with regard to symptomatic sera samples (p < 0.05).

Finally, we compared the diagnostic performance of the current protocol recommended by the Brazilian Ministry of Health to an alternate protocol that replaces the EIE-LVC test developed by Bio-Manguinhos with rLci5 ELISA. While the current protocol offered 59% sensitivity, 98% specificity and an overall accuracy of 80%, the altered protocol presented higher sensitivity (71%), specificity (99%) and accuracy (86%) (Table 4).

**Table 4:** Comparison of diagnostic performance comparing the current Brazilian CVL diagnostic protocol to an alternate protocol employing rLci5 ELISA.

Diagnostic tests		Sensitivity (IC 95%)	Specificity (IC 95%)	Accuracy
Current protocol	DPP-LVC + EIE-LVC^a^	59% (52–66%)	98% (95–99%)	80 (76–84%)
Altered protocol	DPP-LVC + rLci5 ELISA^a^	71% (64–77%)	99% (97–100%)	86 (83–89%)

^a^ p< 0.001; OR = 4.6 (IC 95% 1.7–15.4) McNemar’s Test

## Discussion

The present study demonstrates the relatively low sensitivity offered by the current protocol recommended by the Brazilian Ministry of Health for CVL diagnosis, versus an alternate protocol implementing an ELISA using rLci5, a recombinant protein of *Leishmania*, in place of the EIE-LVC test.

Previous studies have reported satisfactory performance regarding rLci1 (a protein homologous to members of the cytoplasmic HSP70 family) and rLci2 (fragments of the protein belonging to the kinesin superfamily of motor proteins) for the diagnosis of CVL using different platforms [11–16]. While these proteins demonstrated acceptable performance in our recombinant antigen screening protocol, offering high accuracy (84% for both proteins) and high AUC (0.877 and 0.919 for rLci1 and rLci2, respectively), the rLci5 protein (flagellar member protein) and rLci12 (conserved hypothetical protein) provided superior performance: 92% and 87% accuracy, and 0.934 and 0.929 AUC, respectively. Thus, rLci5 was selected for use in a commercial ELISA kit since this protein offered the best isolated performance of those evaluated. In fact, rLci5 was the only protein that presented significantly better performance (OR = 2.8; 1.06–8.77) in comparison to EIE-LVC.

The ELISA test was chosen as a final platform to rLci5 antigen instead an immunochromatographic platform, since there is a necessity of replacement of EIE-LVC due to some limitations of the test. For example, the difficulty in producing the antigen used in the test (lysate of *Leishmania major*) and the difficulty in produce reproducible lots.

Our validation of the performance of rLci5 in an ELISA using a larger sample indicated higher sensitivity and accuracy (87% and 90%, respectively) compared to both DPP-LVC and EIE-LVC (74% and 84%, 67% and 77%, respectively). Although the present performance parameters for rLci5 are lower than those reported in other studies, this difference may be due to the fact that our serum selection process did not consider previous serodiagnostic results; i.e., samples were included based exclusively on previous parasitological or molecular testing, which stands in contrast to results presented in the literature. Our criterion was based on recommendations by Peixoto et al. [19], who identified methodological problems in many studies evaluating diagnostic tests for CVL. In this systematic review, it is noteworthy that the majority of the evaluated studies used as the selection criterion of the sample panel with previous positive results in serologic assays. Thus, the accuracy of these tests could have been overestimated. The chance of bias decreases by not basing serum selection on previously obtained serological results [19,20], the fact that molecular test results were considered for inclusion could compromise sensitivity, as was observed herein (Table 3). The low rate of detection using serological techniques in the canine population with positive results only in qPCR (n = 28/183, 15%) might reflect the presence of low quantities or even the absence of anti-*Leishmania* antibodies in the sample set. In this population, with only qPCR results positive, the rLci5 ELISA was the technique with the highest sensitivity (42%) (Table 3), increasing the detection of dogs that would not be detected by other serological tests (DPP-LVC and EIE-LVC).

The higher sensitivity in the detection of in otherwise undetected samples by another serological tests, reflects in a good detection rate of asymptomatic dogs, however it was lower than the sensitivity on diagnose symptomatic dogs, in agreement with studies that showed a correlation between the level of antibodies and clinical manifestation [21,22]. These results of lower detection of asymptomatic dogs comparing with symptomatic dogs are consistent with those obtained by Grimaldi et al. [23] using DPP, and Porrozi et al. [24] using recombinant antigen in an ELISA assay. In rLci5 ELISA validation, sensitivity showed an increase between the development phase when rLci5 ELISA was evaluated using a reference sample panel (71%) and validation phase (83%). The sera of asymptomatic dogs in both panels presents positive results for qPCR and culture of spleen aspirate, so this improvement may have occurred due to the optimization of the protocol with the use of an antigen stabilizing and blocking reagent (Well-Champion) and a ready-to-use substrate for colorimetric detection reagent (TMB-Plus), the same reagents used in EIE-LVC.

Evaluating rLci5 protein sequence in GeneDB databases, it was identified that this protein is present in the flagellum of promastigote forms and the flagellar button of amastigotes of *Leishmania* [25]. Better performance of rLci5 in comparison with other antigens may be related to the fact that it is more exposed to the immune system of the host, increasing the chances of antibody production against this protein. The cross-reactivity against sera from dogs infected by *T*. *cruzi* found in the present study (35.5%) could be explained by the fact of this protein be conserved in the genus *Trypanosoma*. However, this cross-reactivity is not a problem in the use of rLci5 ELISA in the diagnostic protocol combined with DPP-LVC since the rLci5 ELISA will be used as the confirmatory test performed in the laboratory and, consequently, only samples detected positive by DPP-LVC in the screening will be evaluated in rLci5 ELISA. The specificity observed in this protocol in our study was 99% using sera from non-infected dogs with *Leishmania* and dogs infected by *T*. *cruzi*. Additionally, our next steps will be the evaluation of the use of synthetic peptides derived from Lci5 in an immunochromatographic test to improve the accuracy and decrease the cross-reactivity of the test. Regarding the performance evaluation of the diagnostic protocol recommended by the Brazilian Ministry of Health, the values of sensitivity and accuracy (59% and 80%) obtained in the present study were lower than the results obtained by Fraga et al. [8] that were 73% and 94% for sensitivity and accuracy, respectively. This difference might have occurred due to the use of latent class as gold standard [8].

In summary, our findings showed that rLci5 ELISA presented a higher performance when compared to EIE-LVC and DPP-LVC and these data presented herein strongly support the idea that the replacement of EIE-LVC by rLci5 ELISA as a confirmatory test in the CVL diagnostic protocol can increase sensitivity and accuracy of the diagnostic protocol recommended by Ministry of Health in Brazil, contributing to improve CVL diagnosis and consequently improving the control of VL in Brazil. Nevertheless, this modification in the CVL diagnostic protocol should only be implemented after a multicentric study using representative samples of different regions in Brazil confirming the results obtained in this study.

## Supporting information

S1 Table STARD checklist. Standards for the Reporting of Diagnostic Accuracy Studies (STARD) checklist for reporting of studies of diagnostic accuracy.
(PDF)

S1 Fig STARD flowchart. Standards for the Reporting of Diagnostic Accuracy Studies (STARD) description of the study design.
(TIF)
