# Correction: High accuracy of an ELISA test based in a flagella antigen of *Leishmania* in serodiagnosis of canine visceral leishmaniasis with potential to improve the control measures in Brazil—A Phase II study

**DOI:** 10.1371/journal.pntd.0008346

**Published:** 2020-05-20

**Authors:** Lairton Souza Borja, Lívia Brito Coelho, Matheus Silva de Jesus, Artur Trancoso Lopo de Queiroz, Paola Alejandra Fiorani Celedon, Nilson Ivo Tonin Zanchin, Edimilson Domingos Silva, Antônio Gomes Pinto Ferreira, Marco Aurélio Krieger, Patrícia Sampaio Tavares Veras, Deborah Bittencourt Mothé Fraga

The sixth author’s name is spelled incorrectly. The correct name is: Nilson Ivo Tonin Zanchin.

The correct citation is: Borja LS, Coelho LB, Jesus MSd, de Queiroz ATL, Celedon PAF, Zanchin NIT, et al. (2018) High accuracy of an ELISA test based in a flagella antigen of Leishmania in serodiagnosis of canine visceral leishmaniasis with potential to improve the control measures in Brazil—A Phase II study. PLoS Negl Trop Dis 12(10): e0006871. https://doi.org/10.1371/journal.pntd.0006871

The authors apologize for the errors in the published article.
