# Supplementary figures and images for: High accuracy of an ELISA test based in a flagella antigen of Leishmania in serodiagnosis of canine visceral leishmaniasis with potential to improve the control measures in Brazil – A Phase II study
Source: PLoS Negl Trop Dis. 2018 Oct 26;12(10):e0006871. doi: 10.1371/journal.pntd.0006871 (PMC6231677; doi:10.1371/journal.pntd.0006871)

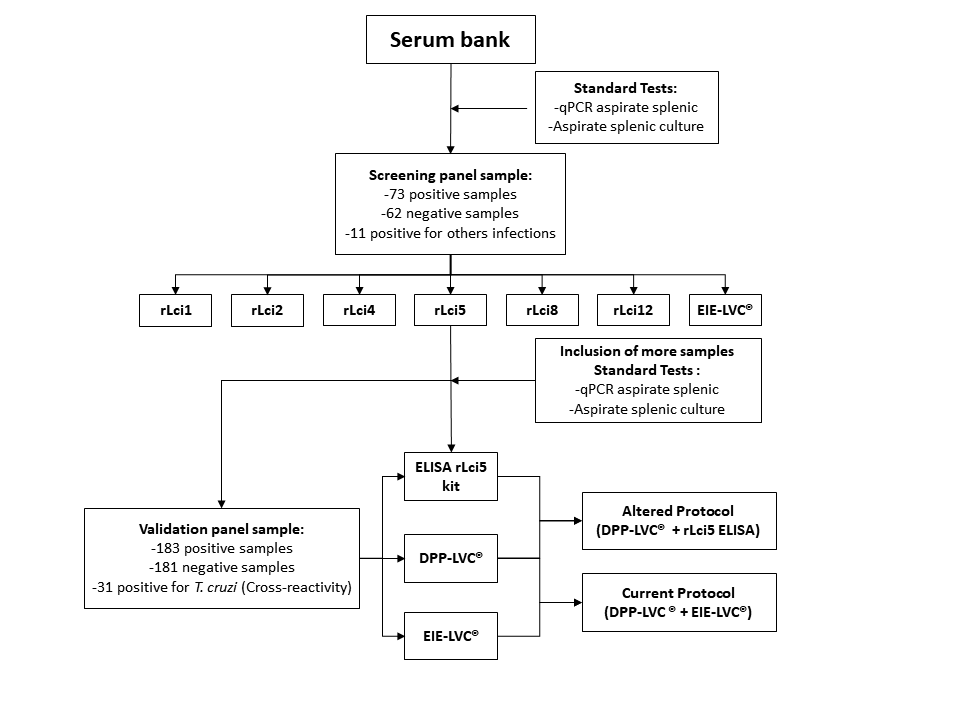

Supplement: S1 Fig — Standards for the Reporting of Diagnostic Accuracy Studies (STARD) description of the study design. (TIF) [file pntd.0006871.s002.tif]
